# Maintenance Chemotherapy Use for Advanced Non-Small Cell Lung Cancer in an Australian Cancer Centre

**DOI:** 10.4021/wjon590w

**Published:** 2013-01-04

**Authors:** Alysson Wann, Genni Newnham

**Affiliations:** a St Vincent’s Hospital, 41 Victoria St, Fitzroy, Victoria 3065, Australia

**Keywords:** Australia, Maintenance, Therapy, NSCLC, Outcomes

## Abstract

**Background:**

To investigate the rates of maintenance therapy in advanced non-small cell cancer, the reasons for not progressing to second line therapy at disease progression at our cancer centre and to use this data as a way to institute it into clinical practice in our cancer centre.

**Method:**

This study was approved by the ethics committee. The data was collected from a purpose built cancer unit database, patient and pharmacy records for all patients diagnosed with Stage 3 and 4 non-small cell lung cancer between 2005 - 2011. Demographic information was collected and subgroup analysis of mean overall survival was obtained. Reasons for not progressing to second line therapy were also analysed.

**Results:**

Of the 105 patients available for analysis, 44 achieved stable disease/partial response (SD/PR) post first cycle of which 42 were eligible for maintenance chemotherapy, 7 went onto receive maintenance with a mean overall survival (OS) of 18.26 months, 23 received second line with the highest OS of 28.19 months and 12 didn’t receive either with the lowest OS of 11.52 months. The majority of these patients did not receive second line at disease progression because of being too unwell.

**Conclusion:**

Similar data on the progression to second line chemotherapy in this patient group was seen. Those that received second line chemotherapy had higher overall survival and thus maintenance therapy could be a means to allow patients to be fit enough to receive second line when they need it.

## Introduction

Non-small cell lung carcinoma (NSCLC) is a common and lethal disease, accounting for 22% of all cancer deaths in males and 15% in females in Australia [1]. The poor outcomes associated with NSCLC can in part be explained by the fact that 80% of patients present with advanced disease [2]. Despite recent advances in the treatment of NSCLC, outcomes for those with advanced disease remain disappointing, with five year survival figures in the order of 11% to 15% [3]. Treatment aims in these patients include prolongation of survival, symptom control and maintenance of quality of life - all of equal importance.

Recent advances in the treatment of advanced NSCLC include the identification and successful targeting of specific molecular abnormalities such as mutations of the epidermal growth factor receptor (EGFR) [4, 5] and the anaplastic lymphoma kinase (ALK) gene rearrangement [6]. In patients whose tumours harbour the appropriate genetic abnormality, these treatments have been associated with significant clinical and radiographic disease responses, often in the absence of major adverse effects. However, as these molecular abnormalities are only identified in a small percentage of NSCLC, the majority of patients with advanced NSCLC still receive cytotoxic chemotherapy as their first line treatment. Systemic chemotherapy in advanced NSCLC has been associated with significant but modest benefits in a number of trials [7-9]. As well as small survival improvements, chemotherapy has been associated with documented improvements in quality of life [10, 11]. Symptomatic improvement with chemotherapy in advanced NSCLC is usually seen within 2 - 3 cycles of treatment, and tends to correlate with disease response or stable disease on imaging [12].

Traditionally, first line chemotherapy has been administered for a predefined period (usually 4 - 6 cycles), after which patients are observed off treatment until evidence of disease progression, at which time second line treatment options are explored. Available data regarding continuation of first line chemotherapy past 4 cycles documents some improvements in progression free survival but no overall survival advantage [13].This approach is currently not recommended in the American Society of Clinical Oncology guidelines for the treatment of stage IV NSCLC.

However, a significant percentage (66%) of patients is observed after initial first-line therapy does not ever come to receive second line treatment [14]. This observation has prompted investigation into the utility of maintenance therapies. Several approaches to maintenance therapy have been investigated, namely continuation of a single agent that has been part of first line chemotherapy [15] or switching to an alternative cytotoxic [16, 17] or targeted agent [18, 19]. These approaches have been associated with modest improvements in progression free and in some cases overall survival.

The aforementioned studies demonstrated improvements in PFS ranging from 1.2 weeks to 3 months. The use of maintenance erlotinib in the SATURN study was associated with a 2.3 month improvement in overall survival for the subgroup of patients whose best response to first-line chemotherapy was stable disease [18]. Other trials have shown overall survival benefits of 0.8 months [19] and 5.3 months (in non-squamous NSCLC only) [20] with the use of platinum-based and pemetrexed chemotherapy respectively, as maintenance treatment. In a disease where overall survival is measured in months, these small advances may be clinically meaningful, but the place of maintenance treatment needs to be carefully balanced with patient preference and the potential negative effects of extended treatment on quality of life.

Despite a significantly higher incidence of adverse events in those patients receiving erlotinib in the SATURN study when compared to those receiving placebo (65% vs. 20%) no difference was identified in overall quality of life measurements between the two groups [21].

Based on the results of these maintenance studies, the Therapeutic Goods Administration has approved the use of both pemetrexed and erlotinib as maintenance therapies in Australia, and pemetrexed is currently subsidised by the Pharmaceutical Benefits Scheme for this indication.

### AIM

In order to rationalise the place of maintenance therapy in our treatment paradigm for NSCLC, we were interested to ascertain the rates of second-line treatment in our advanced NSCLC patients. In addition, for those patients not proceeding to second-line treatment, we were interested to determine the reason.

With this information, we hoped to establish guidelines for the use of maintenance therapies in advanced NSCLC at our Cancer Centre.

## Method

Approval for this study was granted by the institutional ethics review board. Data was initially extracted from a purpose built cancer database, from which we identified all patients with stages 3B or 4 NSCLC treated at the St Vincent’s Hospital Cancer Centre or satellite clinic between January 2005 and December 2011. When necessary, additional information was obtained from hospital medical records, cancer centre records, correspondence and pharmacy records. All data was analysed in a de-identified manner.

Detailed information was collected including demographic data, histopathology, tumour stage, treatment regimens, and dates of diagnosis, treatment failures and death. Descriptive statistics were used to determine the proportion of patients eligible for maintenance therapy, the number receiving maintenance chemotherapy, and the proportion undergoing observation after first-line therapy. In those who did not receive maintenance therapy we determined the percentage actually proceeding to second-line treatment, and in those not receiving second-line treatment, we interrogated the records to determine why.

## Results

Initial analysis of the database identified 123 patients receiving treatment for advanced NSCLC between 2005 and 2011. Eighteen patients were excluded from further analysis for a variety of reasons (12 were discussed at our multidisciplinary meeting but managed at other centres, 4 did not receive any chemotherapy, 1 received adjuvant radiotherapy and 1 had a diagnosis of adenocarcinoma of unknown primary rather than NSCLC).

The demographics of the remaining 105 patients are shown in Table 1.

**Table 1:** Breakdown of Demographics of Original Cohort

Demographics	Number	Percentage
Age		
< 61	25	24
61 - 74	44	42
> 75	36	34
Gender		
Male	65	62
Female	40	38
Histology		
Adenocarcinoma	44	42
Squamous cell carcinoma	15	14
Large cell carcinoma	23	22
Unknown	23	22

The majority of the patients had adenocarcinoma as their histological diagnosis and 62% of the patients were male.

Details of first-line treatment are included in Table 2. The vast majority of patients were treated with carboplatin and gemcitabine chemotherapy. Two patients were enrolled in the Abigail study [22] of maintenance bevacizumab after first-line platinum-based bevacizumab containing chemotherapy.

**Table 2:** Details of First Line Chemotherapy

CarboGem	Gemcitabine	Abigail	Carbotaxel	Gefitinib	UK
94	5	2	2	1	1

Breakdown of first line chemotherapy regimes.

At the conclusion of first-line therapy, 49/105 (47%) patients had progressive disease, 33/105 (31%) patients had stable disease (SD) and 11/105 (11%) showed a partial response (PR) to therapy. The rest of the patients 12/105 (11%) were not assessed or had response that were unknown from the available data. Of the 44 (42%) patients with either responding or stable disease, 42 were eligible for maintenance treatment and 2 were considered unfit at the completion of first-line chemotherapy.

Seven patients with non-progressive disease after first-line treatment went on to receive maintenance therapy (or placebo) in the context of clinical trials: 2 on the Abigail study [22] and 5 on the Saturn study (maintenance erlotinib or placebo after platinum-based chemotherapy) [18]. The duration of maintenance treatment ranged from 1 - 4 months in patients receiving erlotinib/placebo, and 15 - 19 months in those receiving bevacizumab.

The mean overall survival (OS) for those that received maintenance therapy (although the numbers were too few to be of statistical significance) was 18.26 months.

Those on maintenance all went on to receive 2nd line chemotherapy at disease progression: out of the 2 patients who were in the Abigail Trial 1 had Gefitinib and the data on the other patient was missing. Out of the 5 patients on the SATURN trial, 1 had Pemetrexed, 2 had Erlotinib and 2 had missing data.

Out of the 42 eligible patients 35 did not receive maintenance and were observed until disease progression prior to further treatment. Of those only 66% (n = 23) actually received 2nd line chemotherapy (Table 3) with pemetrexed being the most commonly used treatment.

**Table 3:** Details of Second Line Chemotherapy

Breakdown of second line chemotherapy regimes					
Pemtrexed	Gefitinib	Vandetanib	Erlotinib	Docetaxel	Vinorelbine
11	4	3	2	2	1

Breakdown of second line chemotherapy regimes.

**Figure 1:**
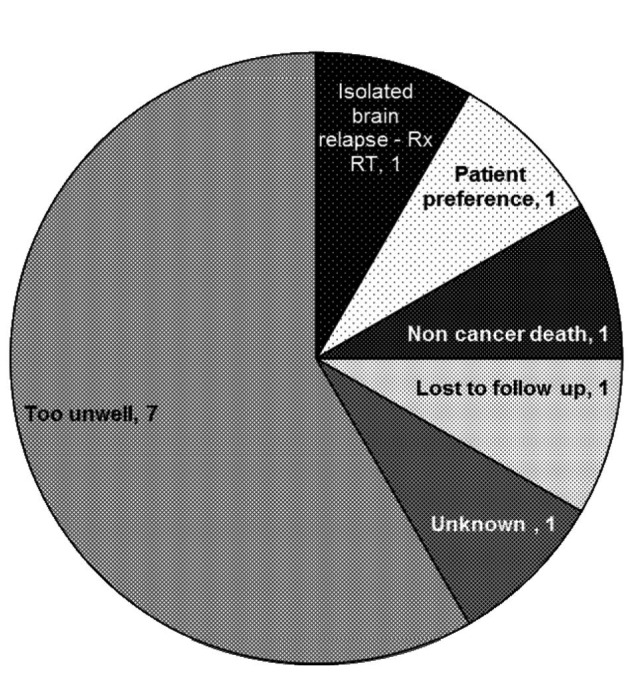
Reasons for not progressing to second line chemotherapy.

The 34% (n = 12) that did not proceed to 2nd line chemotherapy were of particular interest to us. The most common reason for not proceeding to second-line treatment was clinical deterioration (of note, 2 of 7 patients deemed too unwell for second-line treatment were also deemed too unwell for maintenance treatment after first-line chemotherapy and were not included in this analysis), however other reasons included patient preference, non cancer death and isolated brain relapse (Fig. 1)

The mean OS of those that did receive second line therapy was 28 months. As expected the mean OS for those patients that did not receive either maintenance or second line therapy was significantly less at 11 months.

**Figure 2:**
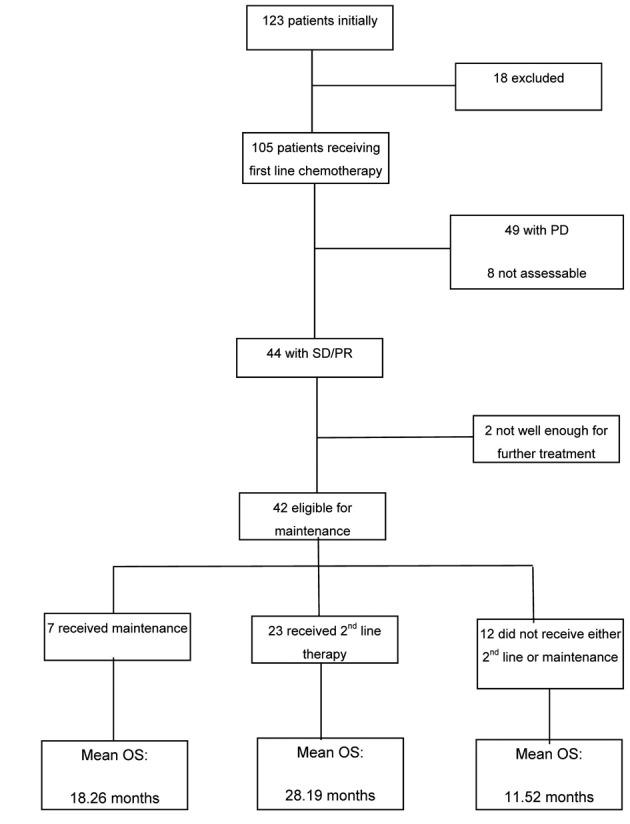
Summary of breakdown of patients and the mean overall survival in the subgroups.

A complete breakdown of all participants with the mean OS for the different subgroups is provided in Figure 2.

## Discussion

The evidence in favour of maintenance chemotherapy in advanced NSCLC is mounting. As yet, the ideal maintenance treatment remains unclear, with evidence of benefit from several different approaches. Two studies have demonstrated positive results by delivering maintenance chemotherapy with an agent that had been used as part of first line chemotherapy. The PARAMOUNT trial [15] demonstrated a small but statistically significant improvement in median PFS (3.9 vs. 2.6 months) with the use of maintenance pemetrexed after initial treatment with cisplatin and pemetrexed. Perol et al [16] randomised responding or stable patients to maintenance with gemcitabine or erlotinib, or observation, after initial treatment with 4 cycles of cisplatin and gemcitabine. Maintenance gemcitabine was associated with improved PFS (3.8 vs. 1.9 months), and a trend to improved OS.

Two studies report improved outcomes by delivering maintenance treatment with a new non cross-resistant cytotoxic agent. Fidias et al [17] investigated the use of single agent docetaxel (up to 6 cycles) in patients with responding or stable disease after 4 cycles of carboplatin and gemcitabine chemotherapy. Patients randomised to receive maintenance docetaxel had longer PFS when compared to those randomised to receive docetaxel only on evidence of disease progression (5.7 vs. 2.7 months). Maintenance docetaxel did not adversely affect quality of life (QOL), and did not significantly alter OS. Of note, only 63% of those randomised to delayed chemotherapy actually received second line treatment, a similar proportion to that seen in our cohort. Ciuleanu et al randomised 663 patients with stable or responding NSCLC after 4 cycles of platinum-containing chemotherapy to treatment with pemetrexed or placebo [20]. After pre-planned subgroup analyses they found that the benefits of this approach were confined to those with non-squamous histology, with improvements in both PFS (4.5 vs. 2.6 months) and OS (15.5 vs. 10.3 months).

The SATURN trial showed the use of EGFR TK inhibitors sequentially in patients with stable or responding NSCLC after first line cytotoxic chemotherapy improved PFS and OS [18]. In this study patients with an objective response or stable disease after 4 cycles of platinum based chemotherapy were randomly assigned to treatment with erlotinib or placebo. Median PFS in the erlotinib group was improved by just over 1 week compared to those receiving placebo (12.3 vs. 11.1 weeks). Whilst improvements in PFS were seen in both adenocarcinoma and squamous cell carcinoma, the magnitude of improvement in PFS was significantly greater in those with EGFR mutation (45 vs. 13 weeks). Improved OS (HR 0.72, P = 0.002) was also seen in another trial with the SATURN investigators, but only in those with stable disease in response to first line chemotherapy (as opposed to partial or complete response) [23]. Erlotinib treatment did not adversely affect quality of life.

The ATLAS trial [24] randomised stable or responding patients to maintenance with either erlotinib or placebo in conjunction with bevacizumab after 4 cycles of platinum based chemotherapy with bevacizumab. The addition of erlotinib to maintenance bevacizumab was associated with improved PFS (4.8 versus 3.8 months). Overall survival data is not yet mature.

A Japanese study [19] of maintenance gefitinib after 3 - 6 cycles of platinum-based chemotherapy revealed small improvements in median PFS in those receiving gefitinib (4.6 vs. 4.3 months). No significant differences were seen in OS when analysing the group as a whole, however statistically significant improvements in the order of 1 month (13.7 vs. 12.9 months) were seen in patients with adenocarcinoma.

Finally, in a French multicentre study that randomised patients to maintenance with either gemcitabine, erlotinib or observation, median PFS was significantly improved in those receiving maintenance when compared to those on observation (erlotinib 2.9 vs. 1.9 months, gemcitabine 3.8 vs. 1.9 months) [16]. A non-significant trend to improved OS was seen with both maintenance arms.

The low rates of maintenance chemotherapy use off study in our cohort can be explained largely by the fact that this data is taken from patients treated between 2005 and 2011, and TGA approval of maintenance pemetrexed was only granted in November 2010. None-the-less, no patients received maintenance chemotherapy off study between November 2010 and the end of Dec 2011. This may relate to several factors including hesitation on the part of treating oncologists for maintenance treatment and patient reluctance to have continued treatment. Our data reveals that almost 55% of patients did go on to receive second-line treatment on disease progression. As described by others, the most common reason for patients not to proceed to second-line treatment in our cohort was clinical deterioration.

Latest overseas data suggest the rate of progression to second line chemotherapy is in the order of 44% [14].

It is interesting to observe survival rates in the various groups however our retrospective analysis is not powered to draw any conclusions relating to survival.

Whilst the use of maintenance chemotherapy in patients with advanced NSCLC is a growing practice, it remains unclear as to which is the preferred agent. For some patients the choice seems relatively clear (for example, erlotinib in those with EGFR mutant NSCLC whose best response to first-line chemotherapy was stable disease and in those with squamous cell carcinoma). For others the choice is more difficult, although simplified somewhat by current PBS restrictions in Australia. It is likely that direct comparisons of maintenance agents will not be made.

In addition, the role of bevacizumab in the treatment of NSCLC, either as part of first-line or maintenance treatment is uncertain. Despite evidence of survival improvements with bevacizumab as part of first-line chemotherapy, the small magnitude of those benefits and the associated increase in toxicity, as well as prescribing restrictions, has meant that it is not often a part of treatment for NSCLC in Australia [25]. As such, the role of bevacizumab in continuation maintenance is unclear.

It is likely that our use of maintenance chemotherapy in patients with advanced NSCLC will increase in response to the increasing supportive data. It is also likely however, that a proportion of patients will elect not to pursue maintenance treatment and opt for a break from active therapy. In these patients it would be prudent to institute a vigilant follow-up process so as to avoid missing the window of opportunity for second-line therapy.
